# Towards a benchmark of abdominal CT use during duty shifts: 15-year sample from the Netherlands

**DOI:** 10.1007/s00261-020-02818-7

**Published:** 2020-10-19

**Authors:** Iliana V. Mengou, Derya Yakar, Ömer Kasalak, Thomas C. Kwee

**Affiliations:** Medical Imaging Center, Department of Radiology, Nuclear Medicine and Molecular Imaging, University of Groningen, University Medical Center Groningen, Hanzeplein 1, P.O. Box 30.001, 9700 RB Groningen, The Netherlands

**Keywords:** Abdomen acute, Incidental findings, Negative results, Night shift work, Spiral CT

## Abstract

**Purpose:**

To investigate temporal changes in the utilization and patient impact of abdominal CT during duty shifts in the past 15 years.

**Methods:**

This study included a random sample of 1761 abdominal CT scans that were made during evening and night duty shifts in a tertiary care center between 2005 and 2019.

**Results:**

The number of CT scans significantly increased (almost threefold) between 2005 and 2019 (Mann–Kendall tau of 0.829, *P* < 0.001). The proportion of negative CT scans (i.e., the absence of findings related to the reason that the CT scan was made and no disease deterioration or other new and clinically relevant findings compared to a previous imaging examination when available) was 40.0% (700/1749) in the entire 15-year study frame and did not significantly change over time (Mann–Kendall tau of − 0.219, *P* = 0.276). The overall frequency of same-day hospital discharge after negative CT was 20.6% (150/729) in the past 15 years and showed a significant increase over time (Mann–Kendall tau of 0.505, *P* = 0.010). The overall proportion of CT scans with incidental findings was 3.4% (60/1761) and remained statistically stable over the past 15 years (Mann–Kendall tau of − 0.057, *P* = 0.804).

**Conclusion:**

Over the past 15 years, the number of CT scans and the frequency of same-day hospital discharge after negative CT have increased, while the proportions of negative CT scans and incidental findings have remained stable in our tertiary care center. The data from this study can be used for interinstitutional benchmarking to define, monitor, and improve the appropriateness of imaging utilization.

## Introduction

Medical imaging plays a crucial role in personalized medicine [1]. Not surprisingly, the number of medical imaging examinations has increased considerably in the Western world over the past two decades, and this also applies to the utilization of computed tomography (CT) [2, 3]. The growth in medical imaging has yielded unarguable benefits to patients in terms of longer lives of higher quality [2]. However, some part of the growth may be attributed to the overutilization of imaging services [4]. Inappropriate imaging utilization puts unnecessary pressure on human and financial healthcare resources, and may harm patients [4].

Negative imaging examinations can be defined as the absence of findings that are related to the reason the study was made and no disease deterioration or other new and clinically relevant findings compared to a previous study when available. For example, a CT scan in a patient with abdominal complaints and the clinical suspicion of appendicitis but without any imaging findings that can explain the symptoms, and a CT scan in another patient with an already known intra-abdominal abscess and the clinical suspicion of abscess growth but with stable imaging findings, can both be considered as negative imaging examinations. Exceedingly high proportions of negative imaging examinations reflect imaging overutilization. Benchmarks have to be established for the acceptable proportion of negative studies [5]. Such benchmarks may be used as key performance indicators of healthcare quality. Alternatively, a negative CT may be used to discharge patients from hospital and thereby save hospitalization costs. Therefore, it is also important to consider the proportion of same-day hospital discharges after a negative CT.

It is hypothesized that along with the increase in imaging utilization over the past years, the proportion of negative studies has also grown. This hypothesis is also thought to apply to the use of abdominal CT scans during evening and night duty shifts. Imaging may also detect findings that are unrelated to the reason the study was ordered. Incidental findings may cause unnecessary additional examinations, interventions, and treatments (also known as the “cascade effect”) [6, 7]). Along with the proportion of CT scans with negative findings and the proportion of same-day hospital discharges after negative CT, data on the proportion of CT scans with incidental findings are relevant to weigh the pros and cons of abdominal CT during evening and night duty shifts.

The purpose of this study was to investigate temporal changes in the utilization and patient impact of abdominal CT during duty shifts in the past 15 years in terms of negative scan proportion, same-day hospital discharge, and incidental findings.

## Materials and methods

### Study design

This study was approved by the local institutional review board and the requirement for informed consent was waived. The University Medical Center Groningen is a tertiary care institution that provides healthcare services to more than 2 million people in the north-east of the Netherlands. Evening and night duty shifts for all radiological subspecialties are covered by a resident in radiology from 17.00 until 8.00 the next day. Residency programs in radiology take 5 full-time years in the Netherlands, and residents are eligible to perform duty shifts after completing the first year of residency. All duty shifts are supervised by staff radiologists. A resident can contact one of the assigned radiologists for immediate supervision during his or her shift, but the resident is not obliged to do so if he or she feels confident to independently perform a particular study. Residents make preliminary reports which are used by requesting physicians to make clinical decisions. All reports are checked and co-signed by a radiologist within 24 h. The Random Calendar Date Generator was used to randomly sample 102 unique calendar dates in one year (representing approximately 27.9% of all days in one year, which matched the minimum sample percentage that was set at 25% by the authors before starting this study) [8]. All abdominal CT scans that were made during evening and night duty shifts on these 102 calendar days in each of the years from 2005 to 2019 (representing 15 consecutive years with a total of 1530 days) were potentially eligible for inclusion. CT scans were included if performed for clinically urgent reasons and if the scan volume included the area from liver dome to pubic symphysis. CT scans were excluded if performed for interventional or therapeutic planning purposes, if performed for non-urgent logistic reasons during duty hours, and if the radiology report and patient files were not available.

### Data extraction

A research fellow (I.V.M.) scrutinized all CT scans and electronic patient files, and applied the aforementioned in- and exclusion criteria. For all CT scans that were finally included, the following parameters were extracted: patient age and gender, requesting department, indication for CT scanning (i.e., acute bowel pathology [obstruction, ischemia, perforation, anastomotic leakage, etc.]; acute oncology; infection or inflammation; non-traumatic vascular; trauma; urolithiasis; or other/mixed), type of CT scan (i.e., unenhanced, arterial, and/or venous phase scan, and use of oral and/or rectal contrast agents), if CT scanning of other body regions was also done in the same session, and if hospital discharge was carried out within 24 h after CT scanning (note that same-day hospital discharges were considered to be within 24 h after CT acquisition, and not by calendar day).

### Data analysis

Based on the original radiology reports, a consensus panel of a research fellow (I.V.M.) and three radiologists (D.Y., Ö.K., T.C.K.) determined for each CT scan if it was positive (i.e., findings that were related to the reason the CT scan was made) or negative (i.e., the absence of findings related to the reason that the CT scan was made and no disease deterioration or other new and clinically relevant findings compared to a previous imaging examination when available). CT scans that could not be certainly categorized as positive or negative were classified as indeterminate. Similarly, for each CT scan, it was determined if it contained an incidental finding (i.e., a finding unrelated to the purpose of the CT scan). Both findings that could result in mortality or considerable morbidity if they were not appropriately treated, and findings for which the effectiveness of intervention or treatment is currently unknown, were considered incidental findings. Predefined presumed benign findings such as simple liver and renal cysts, cholecystolithiasis, and colonic diverticulitis (described in more details by Kwee and Kwee [6]), were not considered clinically significant and were therefore not counted as an incidental finding. Incidental findings that were already known from previous imaging examinations were not counted either. All CT scans were reviewed by the research fellow and at least one of the three radiologists. Although the far majority of decisions on whether the CT scan was positive or negative, and whether or not it contained an incidental finding, appeared to be straightforward, the second and third radiologists were consulted in case of doubt. The final decision was then determined based on the majority vote of the three radiologists. The research fellow was not blinded to any of the parameters that were mentioned in the previous paragraph, whereas all three radiologists were only blinded to the time of hospital discharge. All CT scans were reviewed within 20 consecutive weeks.

### Statistical analysis

The total number of CT scans, the frequency of CT scans with negative findings as a proportion of the total number of CT scans, and the frequency of same-day hospital discharge after CT were calculated for each year from 2005 to 2019. CT scans that were classified as indeterminate with regard to positive or negative findings, were excluded from the proportion calculations. The frequency of CT scans with at least one incidental finding as a proportion of the total number of CT scans was also calculated for each year from 2005 to 2019. Temporal changes were assessed using the Mann–Kendall test. A chi-square test was performed to compare the frequency of same-day hospital discharge after negative CT versus that after positive CT. Logistic regression analyses were performed to determine the association between a negative CT scan and the following variables: patient age and gender, requesting department, indication for CT scanning, and whether or not CT scanning of other body regions was also done in the same session. *P*-values less than 0.05 were considered statistically significant. Statistical analyses were performed using R version 3.6.3 software (R Foundation for Statistical Computing) and MedCalc Statistical Software version 18.5 (MedCalc, Ostend, Belgium).

## Results

### CT scans and patients

**Fig. 1:**
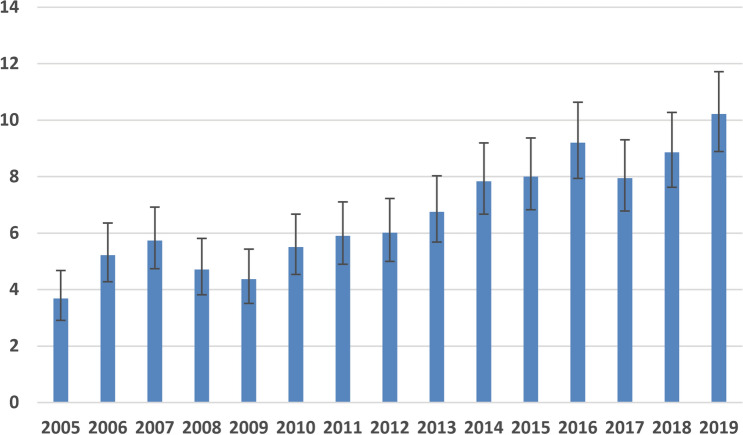
Percentage of abdominal CT scans made during evening and night duty shifts on the 102 randomly selected calendar days for each of the years between 2005 and 2019, as a proportion of the total sample of 1761 abdominal CT scans that were included in this study, along with 95% confidence intervals

Cumulatively, 1861 abdominal CT scans were made during the evening and night duty shifts on the 102 randomly selected calendar days in the years 2005 to 2019. The distribution of these randomly selected calendar days was as follows: January: 11.1% of days; February: 8.3% of days; March: 8.3% of days; April: 0.0% of days; May: 5.6% of days; June: 22.2% of days; July: 5.6% of days; August: 5.6% of days; September 8.3% of days; October: 11.1% of days; November: 2.8% of days; December: 11.1% of days. Excluded CT scans included CT scans that were performed for non-urgent logistic reasons during duty hours (*n* = 70), CT scans that were performed for interventional or therapeutic planning purposes (*n* = 26), and non-availability of the radiology report and patient files (*n* = 4). Eventually, 1761 abdominal CT scans were included, corresponding to an average of 1.15 acute abdominal CT scans per evening and night duty shift over the entire 15-year study period. It should be emphasized that these 1761 abdominal CT scans reflect the data sample and not the absolute true number of scans performed over the time frame. The number of CT scans had increased almost threefold between 2005 and 2019 (Fig. 1), and this increase over the years was significant (Mann–Kendall tau of 0.829, *P* < 0.001). Mean patient age ± SD was 55.4 ± 19.1 years (range 2–107 years), and male/female distribution was 1047/714 at the time of CT scanning. Top-three requesting departments were emergency medicine (996/1762, 56.6%), surgery (306/1762, 17.4%), and internal medicine (259/1762, 14.7%) (Table 1). Top-three indications for CT scanning were other/mixed (447/1762, 25.4%), non-traumatic vascular (436/1762, 24.8%), and infection/inflammation (385/1762, 21.9%) (Table 1). Most CT scans were performed in the venous phase (690/1762, 39.2%) and without oral or rectal contrast agents (1077/1762, 60.9%) (Table 1).

**Table 1:** Summary data on requesting departments, indications for CT scanning, use of intravenous, oral, and rectal CT contrast agents, in order of decreasing frequency (absolute numbers are given with percentages as proportions of the total sample in each category between parentheses)

Requesting department	
Emergency medicine	996 (56.6%)
Surgery	306 (17.4%)
Internal medicine	259 (14.7%)
Intensive care	109 (6.2%)
Gynecology and obstetrics	37 (2.1%)
Urology	23 (1.3%)
Pediatrics	21 (1.2%)
Orthopedics	4 (0.2%)
Unknown	4 (0.2%)
General practitioner	2 (0.1%)
Indication	
Other/mixed	447 (25.4%)
Non-traumatic vascular	436 (24.8%)
Infection or inflammation	385 (21.9%)
Trauma	205 (11.6%)
Acute bowel pathology	190 (10.8%)
Urolithiasis	67 (3.8%)
Acute oncology	31 (1.8%)
Use of intravenous CT contrast agents	
Venous phase	690 (39.2%)
Multiple phases	661 (37.5%)
Arterial phase	287 (16.3%)
Unenhanced	123 (7.0%)
Use of oral and rectal CT contrast agents	
No oral or rectal contrast agents	1077 (60.9%)
Oral contrast agent only	489 (27.8%)
Both oral and rectal contrast agents	183 (10.4%)
Rectal contrast agent only	16 (0.9%)

### Negative CT scans

**Fig. 2:**
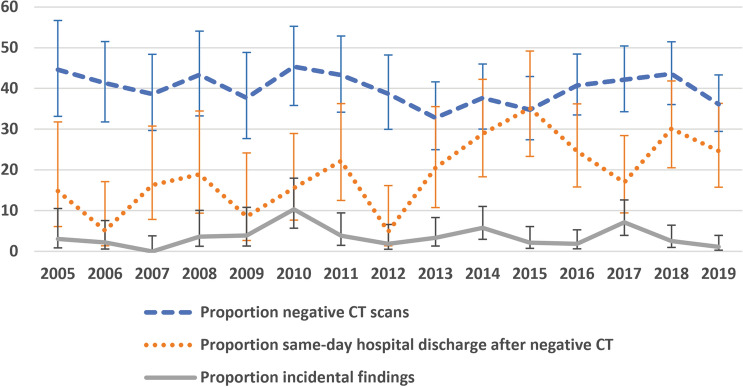
Proportions of CT scans with negative findings, proportion of negative CT scans that were followed by same-day hospital discharge, and proportion of CT scans with incidental findings (%) between 2005 and 2019, along with 95% confidence intervals

Twelve of 1761 CT scans were excluded as they were classified as indeterminate with regard to positive or negative study findings. The proportion of negative CT scans was 40.0% (700/1749) in the entire 15-year study frame. The annual proportions of CT scans with negative findings did not significantly change over time (Mann–Kendall tau of − 0.219, *P* = 0.276) (Fig. 2).

### Same-day hospital discharge

The frequency of same-day hospital discharge after negative CT was 20.6% (150/729) in the entire 15-year study frame, and this was significantly higher (*P* < 0.001) than the same-day hospital discharge frequency of 5.3% (54/1,022) after positive CT. The frequency of same-day hospital discharge after negative CT showed a significant increase over time (Mann–Kendall tau of 0.505, *P* = 0.010) (Fig. 2).

### Incidental findings

The proportion of CT scans with incidental findings was 3.4% (60/1,761). Top-three locations of incidental findings were the adrenal gland (*n* = 19), liver (*n* = 17), and kidney (*n* = 9). The annual proportions of CT scans with incidental findings did not significantly change over time (Mann–Kendall tau of − 0.057, *P* = 0.804) (Fig. 2).

### Association between a negative CT scan and clinical variables

On univariate analysis, there were no significant associations between a negative CT scan and any of the clinical variables (patient age and gender, requesting department, indication for CT scanning, and whether or not CT scanning of other body regions was also done in the same session) (Table 2). Therefore, no subsequent multivariate analysis was done.

**Table 2:** Results of (univariate) logistic regression analysis on the association between a negative CT scan and several clinical variables

Variable	Univariate analysis		
	Odds ratio	95% CI	*P*-value
Patient age	1.003	0.998–1.008	0.289
Patient gender (male vs. female)	0.983	0.810–1.194	0.865
Requesting department	^a^	^a^	0.346
Indication for CT scanning	^a^	^a^	0.368
CT scanning of other body regions (yes vs. no)	1.071	0.875–1.312	0.505
Hospital discharge within 24 h after CT scanning (yes vs. no)	0.967	0.726–1.287	0.812

^a^Because this variable involved multiple categories and the overall model fit was not significant, odds ratios and 95% CIs were not listed for these variables

## Discussion

The results of this study show that the number of abdominal CT scans performed during evening and night duty shifts has increased considerably (approximately threefold) over the past 15 years, which is in line with international temporal trends on the utilization of medical imaging [2, 3]. Overall, 40% of CT scans had negative findings. Given the lack of previous literature on this topic in this setting, it remains unclear if this negative scan rate can be considered acceptable or whether it qualifies as overutilization. The data generated in the present study provide a starting point for future investigation towards the establishment of national and international reference values. Importantly, the annual proportions of negative CT scans did not show any significant changes over the past 15 years, which contradicts our a priori hypothesis that imaging overutilization has increased over time. These results seem to suggest that the overall appropriateness and clinical value of acute abdominal CT imaging did not decline along with the increasing use of this technology in this setting.

Not surprisingly, patients with a negative CT were significantly more frequently discharged on the same day than patients with a positive CT. Interestingly, there was a significant temporal trend towards more same-day hospital discharges after negative CT over time. The reason for this observation remains unclear, but it can be speculated that clinicians increasingly rely on the CT result. It should be noted that a negative CT may prevent hospital admission or continued hospitalization with associated costs. Therefore, based on the temporal stability of the proportion of negative CT scans, and the increased frequency of same-day hospital discharge after negative CT over time, it can be argued that the increased use of abdominal CT during shift hours may even have added value to healthcare. However, whether or not the ordering of these CT scans that turned out to be negative was appropriate in the first place, remains unknown. Judging the appropriateness of imaging utilization is a complicated matter that was beyond the scope of the present study. The data generated by this study should rather be used as a benchmark towards that purpose. In addition, it should be realized that there are many other different factors than the CT result which may influence hospital discharge, including the patient’s clinical condition and the type of health care system. The influence of these and other variables should be investigated by further prospective studies.

Interestingly, none of the investigated clinical variables was associated with a negative CT scan. Further research is necessary to identify sources that carry a higher risk of yielding negative CT scan results.

Overall, 3.4% of CT scans contained an incidental finding and this percentage remained statistically stable over the past 15 years. This frequency can be used by radiologists and clinicians to weigh the potential benefits against the disadvantages of using acute abdominal CT imaging during “out of office” hours. It may also be used for patient counseling and informed consent purposes, given the potential medical consequences of detecting an incidental finding.

There are some previous studies that have investigated the proportion of CT scans with negative findings [9, 10]. For example, in a study by Jacobs et al. [9] in a level one trauma center in the United States, 84.7% (441/522) of patients at risk of blunt abdominal injury had a negative abdominal CT scan in the years 1996–1997. In a more recent study by Hansen et al. [10] in another level one trauma center in the United States, 74.3% (367/494) of intermediate level trauma patients had a negative (head, neck, chest, or abdominal) CT scan in the years 2013–2014. However, it is difficult to compare their results to that of the present study, because Jacobs et al. [9] and Hansen et al. [10] only included trauma patients. Furthermore, these studies did not investigate long-term temporal trends, hospital discharge proportions after CT, and determinants of negative CT scans. Such studies outside the trauma setting are also lacking in the current literature. Similarly, although there is substantial previous literature on incidental imaging findings [11, 12], there is a lack of studies that have been performed in a setting similar to that of the present study. A study by Kelly et al. [13] on the use of abdominal CT in the emergency medicine setting reported an incidental finding rate of 12.4%. This relatively higher frequency may be explained by the fact that Kelly et al. [13] exclusively enrolled patients who presented in the emergency department, whereas the present study also enrolled inpatients.

This study has some limitations. First, radiology residents were the first point of contact for referring physicians during evening and night duty shifts. Skill and experience of the radiology residents may have affected their gatekeeping role to approve or reject a request for CT imaging. Similarly, requesting physicians’ skill and experience may have affected their clinical judgment and reasoning to request a CT scan. However, it was impossible to quantify skill and experience of radiology residents and requesting physicians and to take these parameters into account in our data analysis. Nevertheless, this mix of skill and experience from both sides reflects clinical practice. Second, the results of this study are only applicable to acute abdominal CT imaging during evening and night duty shifts. Further research is necessary to establish reference values of the acceptable proportion of negative studies and their impact on patient management in other settings. Third, only a proportion (27.9%) of all calendar days between 2005 and 2019 was used in this study. However, our sample can be considered representative of and extrapolatable to all calendar days because random sampling was used. This is also reflected by the fact that the almost threefold increase in abdominal CT utilization during evening and night duty shifts between 2005 and 2019 that was found in this study, matches the overall increase in CT utilization in our hospital between 2005 and 2017 when considering all calendar days (based on available records). Fourth, further research is also necessary to determine if our findings from a tertiary care center in Europe hold up in other countries and institutions with different societal and hospital cultures. For example, in a survey that was performed among emergency physicians in the United States in 2013, over 85% of respondents believed too many diagnostic tests are ordered in their own emergency departments, and 97% said at least some (mean: 22%) of the advanced imaging studies they personally order are medically unnecessary [14]. The main perceived contributors were reported fear of missing a low-probability diagnosis and fear of litigation [14]. Fifth, this study did not determine if CT scans were reflective of clinical guidelines, because clinical guidelines usually do not define thresholds of prior probability below which a certain diagnosis (and imaging) should not be considered [5]. Sixth, a future study is required to prospectively collect data related to CT utilization, negative fraction, and discharges to improve overall quality for benchmarking.

In conclusion, over the past 15 years, the number of CT scans and the frequency of same-day hospital discharge after negative CT have increased, while the proportions of negative CT scans and incidental findings have remained stable in our tertiary care center. The data from this study can be used for interinstitutional benchmarking to define, monitor, and improve the appropriateness of imaging utilization.
